# Gluten sensitivity enteropathy in patients with recurrent aphthous stomatitis

**DOI:** 10.1186/1471-230X-9-44

**Published:** 2009-06-17

**Authors:** Ramin Shakeri, Farhad Zamani, Rasoul Sotoudehmanesh, Afsaneh Amiri, Mehdi Mohamadnejad, Fereydoun Davatchi, Ali Mohammadi Karakani, Reza Malekzadeh, Farhad Shahram

**Affiliations:** 1 Digestive Disease Research Center (DDRC), Tehran University of medical sciences, Tehran, Iran; 2 Gastrointestinal and Liver Disease Research Center (GILDRC), Iran University of Medical sciences, Tehran, Iran; 3 Rheumatology Research center, Tehran University of medical sciences, Tehran, Iran; 4 Alborz Hospital, Social Security Organization, Karaj, Iran

## Abstract

**Background:**

Gluten sensitive enteropathy (GSE) is an autoimmune enteropathy triggered by the ingestion of gluten-containing grains in susceptible individuals. Recurrent aphthous stomatitis (RAS) may be the sole manifestation of GSE. The aim of this study was to determine the prevalence of gluten sensitivity enteropathy (GSE) in a large group of patients with RAS and assess the efficacy of gluten free diet (GFD) on the improvement of aphthous lesions in those who were diagnosed with GSE.

**Methods:**

Two hundred and forty seven patients with RAS were included. The patients had at least three aphthous attacks per year. Patients were screened by IgA anti-endomysial antibody (EMA), IgA anti tissue transglutaminase (TTG) and serum IgA level. Those with a positive serology underwent endoscopic biopsies of the duodenal mucosa and patients with negative serology were excluded. The diagnosis of GSE was based on a positive serological test and abnormal duodenal histology. For patients with GSE, gluten free diet was recommended.

**Results:**

Six out of 247 RAS patients had positive TTG test alone, and one had positive EMA and TTG. All 7 patients with positive serologic tests underwent duodenal biopsies. Histological findings were compatible with GSE in all of them (Marsh I in four patients, Marsh II in two patients and Marsh IIIB in one another.). The mean age of GSE patients was 27.42 ± 10.56 (range, 13 to 40) years old. They were suffering from RAS for an average duration of 4.5 years. All of the 7 GSE patients had not responded to the routine anti-aphthae medications, including topical corticosteroids, tetracycline and colchicine. Four patients who adhered to a strict gluten-free diet showed noticeable improvement in their aphthous lesions over a period of 6 months.

**Conclusion:**

A significant minority (e.g. 2.83%) of RAS patients have GSE. This could be compared with the 0.9% prevalence of GSE in the general population of Iran. This study suggests that evaluation for celiac disease is appropriate in patients with RAS. Additionally, the unresponsiveness to conventional anti-aphthae treatment could be an additional risk indicator.

## Background

Gluten sensitive enteropathy (GSE) is an autoimmune enteropathy triggered by the ingestion of gluten-containing grains in susceptible individuals. The presentations of GSE vary clinically, from atypical (without gastrointestinal symptoms), silent and latent to severe forms with gastrointestinal and neurological complications. Although GSE had been identified mainly in individuals of European descent [1], recent data suggests that celiac disease is a common disorder, not only in populations of European ancestry, but also in developing areas, such as North Africa, Middle East and India [2].

Over the past decades, our knowledge on GSE has improved, and many silent or latent cases of GSE have been diagnosed by screening with serological tests[3,4]. Early diagnosis of GSE allows for immediate treatment with a gluten free diet, restores health, and might prevent the development of potential complications associated with GSE (e.g. non-Hodgkin's lymphoma of the gut). [5,6].

Recurrent aphthous stomatitis (RAS) is one of the most common mucosal diseases Aphthae can affect either gender at any age, although a higher prevalence is noted among children, adolescents and females [1]. The prevalence of RAS in general population is estimated to be at least 5% [7]. It is one of the important causes of outpatient visits. It has been reported that in 5% of GSE patients, RAS may be the sole manifestation of the disease [8].

The association between CD and RAS has been evaluated in several studies but conflicting results have been reported [9-11]. Therefore, we conducted this study to determine the frequency of gluten sensitivity enteropathy (GSE) in patients with RAS, using relevant serologic as well as histologic tests. We also aimed to assess the efficacy of gluten free diet (GFD) on the improvement of aphthous lesions in those who were diagnosed with GSE

## Methods

Over a period of 24 months, all patients with RAS who attended the Behcet's clinic of Shariati hospital in Tehran were asked to participate in a screening program for GSE. Patients included in the study had at least three episodes of oral aphthae during the year; exclusion criteria were Behcet's disease, inflammatory bowel disease, systemic lupus erythematousis, tumors of oral cavity, Reiter syndrome and oral lesions due to drugs and radiation. Soft tissues examination was carried out with conventional dental chairs, artificial light, flat mirrors, monouse probe and sterile gauzes. We registered, lesions as RAS if they match one of these three conditions: clinically confirmed by physician, referred by patients themselves and reported by hospital clinical records.

The objectives of the study, as well as the possible necessity for a small bowel biopsy, were explained to the patients. Of 290 eligible patients, 247 agreed to participate in our study. Written informed consent was obtained from each participant, an interviewer completed a clinical questionnaire, and five-milliliter venous blood sample was obtained from each patient for serological investigation of GSE.

The study was performed according to the principles of the Digestive Disease Research Center of Tehran University of Medical Science, and was approved by the Ethics Committee of this center and Rheumatology research center of Tehran University of Medical Science.

A complete history of oral aphthous was taken from all subjects regarding number of aphthous ulcers, duration, size, precipitating factor, response to conventional therapy (such as topical corticosteroids, tetracycline and colchicine) and number of attacks per year.

### IgA-Anti endomysial antibody

Serum IgA antibodies against endomysium were measured by the indirect immunofluorescence method using commercial kits (Biosystem Madrid, Spain). Briefly, patient serum is incubated on tissue sections of monkey esophagus to allow binding of antibodies to the substrate. Any antibodies not bound are removed by rinsing. Bound antibodies of the IgA class are detected by incubation of the substrate with fluorescein-labeled, anti-human immunoglobulin conjugate. Reactions are observed under a fluorescence microscope equipped with appropriate filters. The presence of EMA is demonstrated by an apple green fluorescence of the endomysial lining of smooth muscle bundles. The titer (the reciprocal of the highest dilution giving a positive reaction) of the antibody is then determined by testing serial dilutions.

### IgA Tissue transglutaminase antibody

IgA antibodies to tissue transglutaminase were assessed by ELISA using recombinant human tTG as the antigen (Genesis Diagnostics, Cambridgeshire, UK). Serum samples were diluted to 1:100 with distilled water, incubated with antigen for 30 min at room temperature, washed three times, and subsequently incubated for another 30 min with antihuman IgA. Optical density was read at 450 nm. Results were expressed in arbitrary units (AU) according to the reference calibrator. The cutoff value for a positive outcome was considered to be 7 AU, according to the instruction on the kit.

Serum IgA level was measured to rule out IgA deficiency.

### Hematology and biochemistry

Complementary hematologic and biochemical investigations including complete blood count, platelet, erythrocyte sedimentation rate, C-reactive protein, serum sodium, serum potassium, serum calcium, serum phosphorous, blood urea nitrogen, creatinine,, cholesterol, triglyceride, alanine aminotransferase (ALT), aspartate aminotransferase (AST), alkaline phosphatase, total iron binding capacity, ferritin, serum iron, stool guaiac exam (OB), total protein and albumin were performed in patients who had been diagnosed as GSE.

### Duodenal biopsy

Patients with positive serologic test (EMA or tTG) underwent upper endoscopy and four biopsies were taken from the second portion of the duodenum.

Each biopsy was oriented over a filter paper as well as being prepared for standard histological assessment. The histological assessment included examination for significant alteration in main mucosal measurements and lymphocytic infiltration, Based on Marsh criteria [12]:

0: Normal mucosal structure without a significant lymphocytic infiltration

І: Lymphocytic enteritis (more than 30 lymphocytes/100 epithelial cells).

П: Lymphocytic enteritis and crypt hyperplasia.

ШA: Partial villous atrophy.

ШB: Subtotal villous atrophy.

ШC: Total villous atrophy.

### Gluten-sensitivity enteropathy diagnosis

The presence of positive tTG or EMA plus abnormal duodenal histology (Marsh І, П or Ш) was defined as GSE.

### Statistical analysis

Data are presented as Mean ± SD or percentage. Statistical analyses were performed using SPSS software version 15.

## Results

One hundred thirty one (e.g. 53%) out of 247 RAS patients were men. Mean age was 33 ± 11 years old (range, 11 to 66 year old). After serologic studies for GSE, one patient had positive EMA and tTG tests and six patients were only tTG positive (four men and three women). All patients underwent upper GI endoscopy and duodenal biopsies. In two patients endoscopic finding was compatible with GSE (mild villous atrophy) and five patients had normal endoscopic view. Pathological finding was compatible with Marsh I in four patients, Marsh II in two patients and Marsh IIIB in one.

The mean age of patients with GSE was 27 ± 10 (from 13 to 40) with a female/male ratio of 3/4. They were suffering from RAS for an average duration of 4.5 years. All the GSE patients were unresponsive to previous trials of conventional anti-aphthae drugs (e.g. topical corticosteroids, tetracycline and colchicine) (Table 1). Complementary laboratory tests of these patients were normal except mild anemia in two patients. None of them complained from other typical or atypical signs and symptoms of CD.

**Table 1:** Characteristics of subjects with positive serologic antibodies for gluten-sensitive enteropathy

Patient no.	sex	age	Duration of RAS(y)	No. of attacks per year	Response to conventional therapy	serology	Marsh classification	Other symptoms	Improvement with GFD
1	F	13	2	12<	No	tTG +	II	Anemia	Yes

2	M	33	5	12<	No	tTG +	I	No	--

3	F	40	7	6 to12	No	tTG +	I	No	---

4	M	40	6	6 to12	No	tTG +	I	No	----

5	M	18	3	12<	No	AEA+ & tTG+	III	Anemia	Yes

6	M	25	5	12<	No	tTG +	I	No	Yes

7	F	23	4	6 to12	No	tTG +	II	No	Yes

tTG: tissue transglutaminase antibody, EMA: anti endomysial antibody.

Four out of the seven GSE started a strict gluten free diet. One of them had Marsh I, two had Marsh II, and one had Marsh IIIB lesion. All of them showed a significant improvement within 2–6 months after beginning of GFD.

In the four patients, the mean number of attacks of the aphthous lesions was 6.25 ± 0.96 in the last 6 months proceeding to GFD and decreased to 1.5 ± 0.58 over the first 6 months of GFD adherence.

## Discussion

In this study of a large group of patients with RAS, a 2.83% prevalence of GSE was observed, as compared with an estimated prevalence of 0.9% in the general population of Iran [13].

In order to avoid the controversy in the definition of CD, we used the term "gluten sensitive enteropathy" rather than celiac disease to describe patients with any degrees of intestinal damage together with positive serologic tests.

It has been reported that RAS is at least among the fifth commonest presentations of CD [14-16]. Furthermore, oral mucosal lesions or dental enamel defects may be the sole presenting features of celiac disease [17].

Despite detailed investigations, RAS still has an unknown etiology and poorly effective management [18,19]. Genetic, immunological and microbial factors may play a role in the pathogenesis of RAS, whereas attacks may be precipitated by local trauma, stress, food intake, some drugs, hormonal changes or vitamin and trace element deficiencies [19]. RAS can arise in some systemic disorders including: Behcet's disease [20,21], Sweet's syndrome [22,23], MAGIC syndrome [24], inflammatory bowel disease [25,26] and gluten-sensitivity enteropathy (celiac disease) [27].

The association between RAS and gluten-sensitivity enteropathy (GSE) was proposed in 1976 by Ferguson et al [28] when they found 24% of patients with RAS showed histological evidence of CD on jejunal biopsy. Nevertheless, there is still considerable dispute concerning the actual prevalence of CD among patients with RAS, as different studies have reported different prevalence of CD in RAS patients [29-34]. On the other hand in recent years, some articles are published which expressed little or no significant etiological link between RAS and CD, and added that screening RAS patients for key serological markers of CD is of little clinical value [10,1]. Currently, there is no approved recommendation which can be used by clinicians to approach patients with RAS regarding celiac disease. Comparing to the previous studies, this study with a large group of patients and by using two more sensitive and specific serologic tests plus duodenal biopsy helped us reach a reliable conclusion. Prevalence of GSE in patients with RAS was 2.83%, which is about 3-fold higher than that expected in general population of Iran (0.9%) [13].

The effect of gluten-free diet (GFD) on remission of RAS is still uncertain, as dietary withdrawal of gluten occasionally results in significant benefit whereas some studies reported it ineffective [11,35,36]. Four patients accepted to start GFD, and all of them showed a significant improvement within 2–6 months after beginning of GFD. Furthermore anemia resolved after 6 months of follow up in the two patients who suffered from anemia.

Many physicians may still consider the gastrointestinal signs and symptoms as a main manifestation of celiac patients whereas recent studies demonstrated that gastrointestinal presentations may be absent in GSE patients especially in the beginning of the disease. In this study, none of our GSE patients had any gastrointestinal symptoms. Therefore, gastrointestinal symptoms are sometimes absent in the setting of the disease and RAS could be the first or the sole presentation of GSE.

Our study has some limitations. We did not take duodenal biopsies from the patients who had negative serological tests. It has been reported that the sensitivities of the serological tests are decreased in GSE patients with minor mucosal damages [37,38]. We cannot exclude the possibility of missing some GSE patients with negative serological tests and Marsh I/II mucosal lesions (e.g. seronegative GSE). However, a patient with negative serological test and duodenal mucosal lesion may suffer from other disorders like autoimmune enteropathy, giardiasis, common variable immunodeficiency, tropical sprue, peptic duodenitis, Crohn's disease etc. Including such patients (e.g. those with negative serological tests with duodenal mucosal damage) in the spectrum of GSE could increase the rate of false positive results; unless symptomatic and histological improvements are confirmed by gluten free diet. Therefore, in the epidemiological studies, a positive result from a highly specific serological test (e.g. EMA, or tTG) together with any degree of duodenal mucosal lesion provide reasonable criteria for identifying patients with GSE.

## Conclusion

In conclusion, a subset of RAS patients suffers from GSE. GSE should be considered in RAS patients; unresponsiveness to conventional anti-aphthae treatment could be an additional risk indicator. Implementation of GFD may prevent the complications of GSE and effectively treat RAS.

## Competing interests

The authors declare that they have no competing interests.

## Authors' contributions

RS Participated in Conception, design, undertaking study and manuscript writing. FZ Participated in Design, undertaking study and manuscript writing. R S: Participated in Conception and experimental design. AA Participated in Undertaking study, data analysis, manuscript writing. MM Participated in Conception, design, data analysis, manuscript writing. FD Participated in Conception and experimental design. AMK Participated in undertaking study, laboratory works. RM Participated in Conception and experimental design, manuscript writing. FS Participated in Conception and experimental design, manuscript writing. All authors read and approved the final manuscript.

## Pre-publication history

The pre-publication history for this paper can be accessed here:

http://www.biomedcentral.com/1471-230X/9/44/prepub
